# Sequence polymorphism and haplogroup data of the hypervariable regions on mtDNA in Semoq Beri population

**DOI:** 10.1016/j.dib.2018.10.158

**Published:** 2018-11-08

**Authors:** Muhamad Aidil Zahidin, Wan Bayani Wan Omar, Wan Rohani Wan Taib, Jeffrine Rovie Ryan Japning, Mohd Tajuddin Abdullah

**Affiliations:** a Institute of Tropical Biodiversity and Sustainable Development, Universiti Malaysia Terengganu, 21030 Kuala Nerus, Malaysia; b School of Marine and Environmental Sciences, Universiti Malaysia Terengganu, 21030 Kuala Nerus, Malaysia; c Institute for Community Health Development, Universiti Sultan Zainal Abidin, Level 1, Block E, 21030 Kuala Nerus, Malaysia; d National Wildlife Forensic Laboratory, Department of Wildlife and National Park Peninsular Malaysia, KM 10, Jalan Cheras, 56100 Kuala Lumpur, Malaysia

## Abstract

Orang Asli is the aboriginal people in Peninsular Malaysia who have been recognized as indigenous to the country and still practicing traditional lifestyle. The molecular interest on the Orang Asli started when the earliest prehistoric migration occurred approximately 200 kya and entering Peninsular Malaysia 50 kya in stages. A total of three groups of Orang Asli present in Peninsular Malaysia, namely, Negrito also known as Semang, Senoi and Proto Malays. Through records, there is no research has been conducted on mtDNA variations in the Semoq Beri population, one of the tribes in Senoi group. In this report, variations of mtDNA were analysed in the population in Hulu Terengganu as an initial effort to establish the genetic characterisation and elucidating the history of Orang Asli expansion in Peninsular Malaysia. An array of mtDNA parameters was estimated and the observed polymorphisms with their respective haplogroups in comparison to rCRS were inferred respectively. The DNA sequences are registered in the NCBI with accession numbers KY853670-KY853753.

**Specifications table**

**Table:** 

Subject area	Forensic science
More specific subject area	Forensic genetic
Type of data	Tables and figure
How data were acquired	Data were acquired by extracting, amplifying, purifying, sequencing and analysing the target mtDNA region using PureLink^™^ Genomic DNA Mini Kit (Invitrogen, USA), QIAquick Purification Kit (QIAGEN Ag., Germany), DNA sequencer (First Base Laboratories, Malaysia), Sequencher 5.4 software (https://genecodes.com), ClustalW2 MUSCLE (https://www.ecbi.ac.uk), MEGA 7 software [1], DnaSP 5.1 software [2] and Haplogroup software (https://dna.jameslick.com)
Data format	Raw and analysed
Experimental factors	Blood sample collection, DNA extraction, PCR amplification, DNA purification, sequencing and data interpretation
Experimental features	Sequence analysed followed by haplogroup identification
Data source location	Kampung Sungai Berua, Hulu Terengganu, Terengganu, Malaysia
Data accessibility	The mtDNA sequences are registered in the NCBI with accession number KY853670-KY853753 [Table S1]
Related research article	Zahidin [3]

**Value of the data**

• Presently, there are 533 Semoq Beri and likely to be a threatened population in Hulu Terengganu due to the culture assimilation and intermarriage [3], [4], [5], [6].
• The data provide baseline information to any future genetic and evolutionary studies as inferred from control region mtDNA.
• The data will enhance the DNA database of Semoq Beri population to elucidating the history of Orang Asli expansion in Peninsular Malaysia.
• The data allow other researchers focusing on this population to start genome-wide analysis.

## 1 Data

**Fig. 1:**
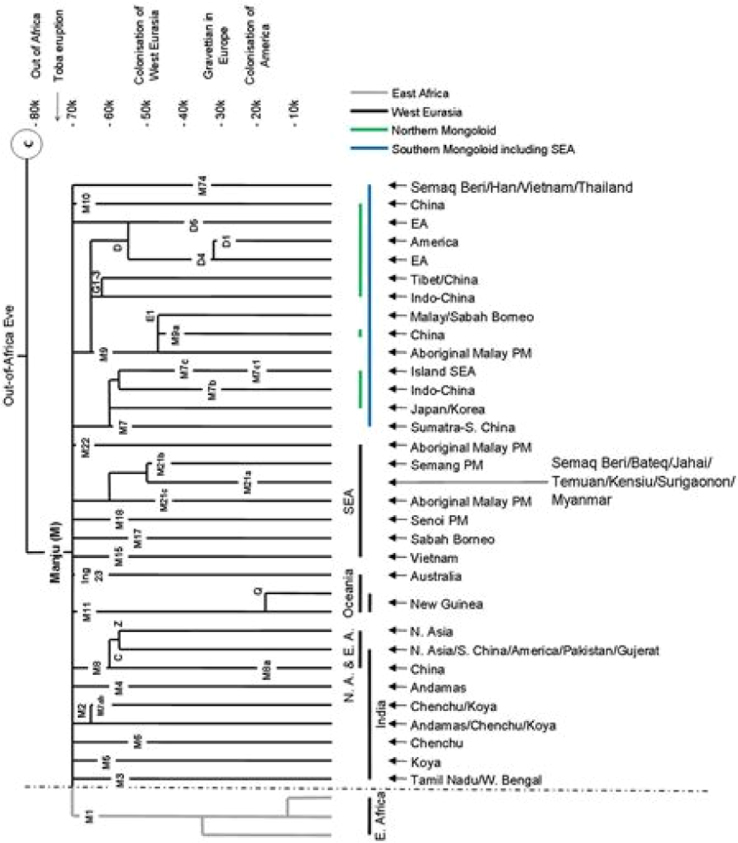
The current Asian and Pacific mtDNA within Manju clan. The tree was reconstructed based on [11]. The uppercase letter (E-East, N-North, S-South, NA-North Asia, EA-East Asia, SEA-Southeast Asia and PM-Peninsular Malaysia) is referring to the geographical location.

**Fig. 2:**
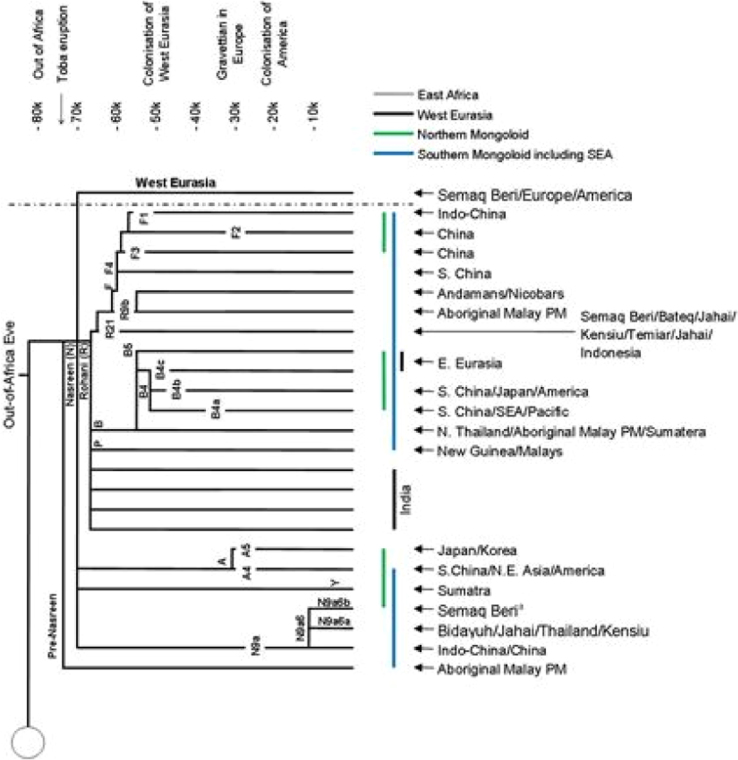
The current Asian and Pacific mtDNA within Nasreen clan. The tree was reconstructed based on [11]. The uppercase letter (E-East, N-North, S-South, NA-North Asia, EA-East Asia, SEA-Southeast Asia and PM-Peninsular Malaysia) is referring to the geographical location.

This data article is possible after unrelated blood samples successfully sequenced as inferred from Hypervariable Segment I (HVSI) and HVSII of mtDNA (Table 1). Each sequence was subjected into Sequencher 5.4 software (https://genecodes.com), ClustalW2 MUSCLE (https://www.ecbi.ac.uk) and MEGA 7 software [1] to identify the sequence polymorphisms (Table S1), C-stretch (Table 2) and nucleotide composition (Table 3). Meanwhile, haplotype data (Table 4) were obtained through DnaSP 5.1 software [2]. Haplogroup classification was performed by using Haplogroup software (https://dna.jameslick.com) on HVSI sequences. The schematic diagrams that represent two major haplogroups, which are M and N, were drawn ( Figs. 1 and 2).

**Table 1:** Details of the primers used for PCR amplification [3].

**mtDNA region**	**Nucleotide position**	**Primers**	**Primer sequences (5′-3′)**	**Size (bp)**
HVI	16,024–16,569	conL1 (F)	TCAAGCTTACACCAGTCTTGTAAACC	600
		conH1 (R)	CCTGAAGTAGGAACCAGATG	
HVII	0–576	conL4 (F)	GGTCTATCACCCTATTAACCAC	600
		conH4 (R)	CTGTTAAAAGTGCATACCGCCA

**Table 2:** C-stretch region of HVII region between nucleotide positions 233 (*233C) to 250 (250C).

**ns**	**2**	**2**	**2**	**2**	**2**	**2**	**2**	**2**	**2**	**2**	**2**	**2**	**2**	**2**	**2**	**2**	**2**	**2**	**n**
	**3**	**3**	**3**	**3**	**3**	**3**	**3**	**4**	**4**	**4**	**4**	**4**	**4**	**4**	**4**	**4**	**4**	**5**	
	**3**	**4**	**5**	**6**	**7**	**8**	**9**	**0**	**1**	**2**	**3**	**4**	**5**	**6**	**7**	**8**	**9**	**0**	
**rCRS**	**N**	**N**	**N**	**C**	**C**	**C**	**C**	**C**	**C**	**C**	**T**	**C**	**C**	**C**	**C**	**C**	**G**	**C**	
24		C	C	C	C	C	C	C	C										8
13			C	C	C	C	C	C	C										7
6	C	C	C	C	C	C	C	C	C										9
1	C	C	C	C	C	C	C	C	C	C	C	C	C	C	C	C			16
1		C	C	C	C	C	C	C	C	C	C	C	C	C	C	C	C	C	17

N - deletion base, ns - total number of sequences, n - total number of unbroken bases C series.

**Table 3:** Sequence variation for the HVI and HVII regions.

**Variation indices**	**HVI region**	**HVII** region
Nucleotide position (%)	16,024 to 16,504 (88%)	72 to 351 (49%)
Base pair	481 bp	280 bp
No. of polymorphic sites	18	26
No. of observed transitions	16	17
No. of observed transversions	2	9
No. of indels	–	5
Nucleotide composition (%) C	31.17	27.66
T	23.74	27.39
A	31.20	28.75
G	13.89	16.20

**Table 4:** Frequency distribution of the mtDNA haplotypes.

	**Haplotype**	**N**	**Samples**	**Frequency**
**HVS-I**	Hap 1	1	Semaq Beri 19	0.025
	Hap 2	2	Semaq Beri 3, 43	0.050
	Hap 3	1	Semaq Beri 45	0.025
	Hap 4	18	Semaq Beri 1, 5, 6, 8, 11, 12, 17, 18, 21, 24, 29, 32, 33, 35, 39, 40, 42, 47	0.450
	Hap 5	7	Semaq Beri 7, 13, 23, 30, 36, 37, 49	0.175
	Hap 6	4	Semaq Beri 20, 27, 28, 34	0.100
	Hap 7	7	Semaq Beri 2, 9, 14, 22, 31, 38, 48	0.175
**HVS-II**	Hap 8	1	Semaq Beri 44	0.023
	Hap 9	1	Semaq Beri 46	0.023
	Hap 10	1	Semaq Beri 21	0.023
	Hap 11	1	Semaq Beri 36	0.023
	Hap 12	1	Semaq Beri 35	0.023
	Hap 13	10	Semaq Beri 2, 9, 14, 20, 22, 27, 28, 31, 38, 48	0.227
	Hap 14	1	Semaq Beri 3	0.023
	Hap 15	1	Semaq Beri 25	0.023
	Hap 16	1	Semaq Beri 26	0.023
	Hap 17	3	Semaq Beri 10, 15, 41	0.068
	Hap 18	2	Semaq Beri 19, 50	0.045
	Hap 19	14	Semaq Beri 1, 5, 6, 8, 11, 12, 17, 18, 24, 29, 32, 39, 40, 47	0.318
	Hap 20	7	Semaq Beri 4, 13, 16, 23, 30, 43, 49	0.159

N - number of haplotype.

## 2 Experimental design, materials, and methods

### 2.1 Sample collection and genomic DNA extraction

All sequence data were generated from DNA samples that were collected with informed and written consent, and approved by Universiti Sultan Zainal Abidin (UniSZA) Human Research Ethics Committee, Malaysia. Blood samples were collected from unrelated individuals of Semoq Beri in Kampung Sungai Berua, Hulu Terengganu, Malaysia. The blood samples were extracted using PureLink^™^ Genomic DNA Mini Kit (Invitrogen, USA) following protocol provided by the manufacturer.

### 2.2 PCR amplification, DNA purification and sequencing

The isolated genomic DNA were amplified using a set of partial forward and reverse HVI and HVII primers respectively (Table 1) [7]. Negative, amplification and reagent blank controls were used to avoid contamination present at any stage during laboratory works. The PCR amplification was carried out in a final volume of 25 μl (Table S2) in Arktik Thermal Cycler (Thermo Scientific, USA) and the PCR profile was given in Table S3. The amplified PCR products were purified using QIAquick Purification Kit (QIAGEN Ag., Germany). The DNA products were visualized using 1% of agarose gel electrophoresis to read the size of the amplified product. The sequencing was carried out at First Base Laboratories Sdn Bhd (Malaysia) using ABI PRISM® 377 DNA Sequencher with the BigDye® Terminator 3.0 Cycle Sequencing Kit.

### 2.3 Statistical sequence analyses

The fluorescence nucleotide bases of segmented DNA sequences were visualized and read using Sequencher 5.4 (https://genecodes.com). The sequences were matched and aligned with the revised Cambridge Reference Sequences (rCRS) [8], [9] using ClustalW2 MUSCLE (Multiple Sequence Comparison by Log-Expectation) (https://www.ebi.ac.uk). The C-stretch for each sequence was checked and counted (Table 2). The nucleotide composition was performed in MEGA 7 [1] (Table 3). The Arlequin haplotype data were generated using DnaSP 5.1 [2] (Table 4). Haplogroup classification was performed using haplogroup online software (https://dna.jameslick.com) where the haplogroup data were compatible with PhyloTree Build 17 [10]. The schematic diagrams were drawn based on [10] and [11] (Figs. 1 and 2). GenBank accession numbers and haplogroups identification for HVI and HVII of Semoq Beri population are provided in Table S1.
