# Supplementary material for: Sequence polymorphism and haplogroup data of the hypervariable regions on mtDNA in Semoq Beri population
Source: Data Brief. 2018 Nov 8;21:2609–15. doi: 10.1016/j.dib.2018.10.158 (PMC6288409; doi:10.1016/j.dib.2018.10.158)
Supplement: Supplementary file 2 — Supplementary Table S1. [file mmc2.doc]

**Supplementary Table 1**

Sequence polymorphism of the HVI and HVII and their respective genbanks and haplogroups in Semoq Beri population.

| **Samples** | **HVI Region (16,024 to 16,504)** | | **HVII Region (72 to 351)** | | **Haplogroup** |
| --- | --- | --- | --- | --- | --- |
|  | **Polymorphism** | **GenBank** | **Polymorphism** | **GenBank** |  |
| Semoq Beri 1 | 16223T 16267A 16271T 16302T 16352C | KY853670 | 73G 150T 263G 305C 306C 313T 314C | KY853710 | N9a6b |
| Semoq Beri 2 | 16168T 16305T 16306T 16314C | KY853671 | 73G 146C 152C 199C 249G 263G 305C 306C 313T 314C | KY853711 | R21 |
| Semoq Beri 3 | 16093C 16129A 16223T 16266T 16281C 16372C 16492G | KY853672 | 73G 146C 263G 305C 306C 313T 314C | KY853712 | M21a |
| Semoq Beri 4 |  |  | 73G 263G 306C 313T 314C | KY853713 |  |
| Semoq Beri 5 | 16223T 16267A 16271T 16302T 16352C | KY853673 | 73G 150T 263G 305C 306C 313T 314C | KY853714 | N9a6b |
| Semoq Beri 6 | 16223T 16267A 16271T 16302T 16352C | KY853674 | 73G 150T 263G 305C 306C 313T 314C | KY853715 | N9a6b |
| Semoq Beri 7 | 16223T 16267A 16321C 16372C | KY853675 |  |  | M74 |
| Semoq Beri 8 | 16223T 16267A 16271T 16302T 16352C | KY853676 | 73G 150T 263G 305C 306C 313T 314C | KY853716 | N9a6b |
| Semoq Beri 9 | 16168T 16305T 16306T 16314C | KY853677 | 73G 146C 152C 199C 249G 263G 305C 306C 313T 314C | KY853717 | R21 |
| Semoq Beri 10 |  |  | 73G 103A 152C 204C 263G 304C 305C 306C 313T 314C | KY853718 |  |
| Semoq Beri 11 | 16223T 16267A 16271T 16302T 16352C | KY853678 | 73G 150T 263G 305C 306C 313T 314C | KY853719 | N9a6b |
| Semoq Beri 12 | 16223T 16267A 16271T 16302T 16352C | KY853679 | 73G 150T 263G 305C 306C 313T 314C | KY853720 | N9a6b |
| Semoq Beri 13 | 16223T 16267A 16321C 16372C | KY853680 | 73G 263G 306C 313T 314C | KY853721 | M74 |
| Semoq Beri 14 | 16168T 16305T 16306T 16314C | KY853681 | 73G 103A 152C 204C 263G 304C 305C 306C 313T 314C | KY853722 | R21 |
| Semoq Beri 15 |  |  | 73G 103A 152C 204C 263G 305C 306C 313T 314C | KY853723 |  |
| Semoq Beri 16 |  |  | 73G 263G 306C 313T 314C | KY853724 |  |
| Semoq Beri 17 | 16223T 16267A 16271T 16302T 16352C | KY853682 | 73G 150T 263G 305C 306C 313T 314C | KY853725 | N9a6b |
| Semoq Beri 18 | 16223T 16267A 16271T 16302T 16352C | KY853683 | 73G 150T 263G 305C 306C 313T 314C | KY853726 | N9a6b |
| Semoq Beri 19 | 16093C 16129A 16223T 16266T 16281C 16372C 16492G | KY853684 | 73G 207A 263G 305C 306C 313T 314C | KY853727 | M21a |
| Semoq Beri 20 | 16168T 16484A 16306T 16314C | KY853685 | 73G 146C 152C 199C 249G 263G 304C 305C 306C 313T 314C | KY853728 | H2a3 |
| Semoq Beri 21 | 16223T 16267A 16271T 16302T 16352C | KY853686 | 73G 146C 152C 199C 263G 306C 313T 314C 321G 333A 349T | KY853729 | N9a6b |
| Semoq Beri 22 | 16168T 16305T 16306T 16314C | KY853687 | 73G 146C 152C 199C 249G 263G 306C 313T 314C | KY853730 | R21 |
| Semoq Beri 23 | 16223T 16267A 16321C 16372C | KY853688 | 73G 263G 306C 313T 314C | KY853731 | M74 |
| Semoq Beri 24 | 16223T 16267A 16271T 16302T 16352C | KY853689 | 73G 150T 263G 305C 306C 313T 314C | KY853732 | N9a6b |
| Semoq Beri 25 |  |  | 73G 103A 152C 204C 263G 305C 306C 313T 314C | KY853733 |  |
| Semoq Beri 26 |  |  | 73G 210G 263G 286A 304C 305C 306C 313T 314C | KY853734 |  |
| Semoq Beri 27 | 16168T 16484A 16306T 16314C | KY853690 | 73G 146C 152C 199C 249G 263G 304C 305C 306C 313T 314C | KY853735 | H2a3 |
| Semoq Beri 28 | 16168T 16484A 16306T 16314C | KY853691 | 73G 146C 152C 199C 249G 263G 304C 305C 306C 313T 314C | KY853736 | H2a3 |
| Semoq Beri 29 | 16223T 16267A 16271T 16302T 16352C | KY853692 | 73G 150T 263G 305C 306C 313T 314C | KY853737 | N9a6b |
| Semoq Beri 30 | 16223T 16267A 16321C 16372C | KY853693 | 73G 263G 306C 313T 314C | KY853738 | M74 |
| Semoq Beri 31 | 16168T 16305T 16306T 16314C | KY853694 | 73G 146C 152C 199C 249G 263G 305C 306C 313T 314C | KY853739 | R21 |
| Semoq Beri 32 | 16223T 16267A 16271T 16302T 16352C | KY853695 | 73G 150T 263G 305C 306C 313T 314C | KY853740 | N9a6b |
| Semoq Beri 33 | 16223T 16267A 16271T 16302T 16352C | KY853696 |  |  | N9a6b |
| Semoq Beri 34 | 16168T 16484A 16306T 16314C | KY853697 |  |  | H2a3 |
| Semoq Beri 35 | 16223T 16267A 16271T 16302T 16352C | KY853698 | 73G 146C 152C 199C 249G 263G 305C 306C 313T 314C 328G | KY853741 | N9a6b |
| Semoq Beri 36 | 16223T 16267A 16321C 16372C | KY853699 | 73G 150T 263G 306C 313T 314C 328G 345C | KY853742 | M74 |
| Semoq Beri 37 | 16223T 16267A 16321C 16372C | KY853700 |  |  | M74 |
| Semoq Beri 38 | 16168T 16305T 16306T 16314C | KY853701 | 73G 146C 152C 199C 249G 263G 306C 313T 314C | KY853743 | R21 |
| Semoq Beri 39 | 16223T 16267A 16271T 16302T 16352C | KY853702 | 73G 150T 263G 305C 306C 313T 314C | KY853744 | N9a6b |
| Semoq Beri 40 | 16223T 16267A 16271T 16302T 16352C | KY853703 | 73G 150T 263G 305C 306C 313T 314C | KY853745 | N9a6b |
| Semoq Beri 41 |  |  | 73G 103A 152C 204C 263G 305C 306C 313T 314C | KY853746 |  |
| Semoq Beri 42 | 16223T 16267A 16271T 16302T 16352C | KY853704 |  |  | N9a6b |
| Semoq Beri 43 | 16093C 16129A 16223T 16266T 16281C 16372C 16492G | KY853705 | 73G 263G 305C 306C 313T 314C | KY853747 | M21a |
| Semoq Beri 44 |  |  | 73G 103A 152C 204C 263G 304C 305C 306C 314C 320T 321T 325G 339T 340T 342A | KY853748 |  |
| Semoq Beri 45 | 16093C 16129A 16223T 16266T 16281C 16372C 16492G | KY853706 |  |  | M21a |
| Semoq Beri 46 |  |  | 73G 103A 152C 204C 263G 305C 306C 314C 320C 328G 330C 333A 342A 347T 349T | KY853749 |  |
| Semoq Beri 47 | 16223T 16267A 16271T 16302T 16352C | KY853707 | 73G 150T 263G 305C 306C 313T 314C | KY853750 | N9a6b |
| Semoq Beri 48 | 16168T 16305T 16306T 16314C | KY853708 | 73G 146C 152C 199C 249G 263G 306C 313T 314C | KY853751 | R21 |
| Semoq Beri 49 | 16223T 16267A 16321C 16372C | KY853709 | 73G 263G 306C 313T 314C | KY853752 | M74 |
| Semoq Beri 50 |  |  | 73G 207A 263G 305C 306C 313T 314C | KY853753 |  |
