# Supplementary material for: Sequence polymorphism and haplogroup data of the hypervariable regions on mtDNA in Semoq Beri population
Source: Data Brief. 2018 Nov 8;21:2609–15. doi: 10.1016/j.dib.2018.10.158 (PMC6288409; doi:10.1016/j.dib.2018.10.158)
Supplement: Supplementary file 3 — Supplementary Table S2. [file mmc3.doc]

**Supplementary Table 2**

Details of the PCR reaction mixture.

| **Reagents** | **Quantity** |
| --- | --- |
| 10X reaction buffer | 2.5 µl |
| 50 mM MgCl_2_ | 1.5 µl |
| dNTP | 0.5 µl |
| Forward primer (conL1) | 1.0 µl |
| Reverse primer (conH1) | 1.0 µl |
| ddH_2_O | 16.3 µl |
| DNA template | 2.0 µl |
| Taq polymerase | 0.2 µl |
| **Total volume** | **25 µl** |
