# Supplementary material for: Sequence polymorphism and haplogroup data of the hypervariable regions on mtDNA in Semoq Beri population
Source: Data Brief. 2018 Nov 8;21:2609–15. doi: 10.1016/j.dib.2018.10.158 (PMC6288409; doi:10.1016/j.dib.2018.10.158)
Supplement: Supplementary file 4 — Supplementary Table S3. [file mmc4.doc]

**Supplementary Table 3**

Details of the PCR amplification profile [3].

| **PCR Profile** | **Temperature** | **Duration** |
| --- | --- | --- |
| Preliminary denaturation | 94 ^o^C | 5 min |
| Denaturation | 94 ^o^C | 30 s |
| Annealing | 54 ^o^C | 30 s |
| Extension | 72 ^o^C | 45 s |
| Final extension | 72 ^o^C | 7 min |
| Soak | 4 ^o^C | ∞ |
