# Supplementary material for: Sequence polymorphism and haplogroup data of the hypervariable regions on mtDNA in Semoq Beri population
Source: Data Brief. 2018 Nov 8;21:2609–15. doi: 10.1016/j.dib.2018.10.158 (PMC6288409; doi:10.1016/j.dib.2018.10.158)
Supplement: Supplementary file 1 — Supplementary material. [file mmc1.docx]

Hao-Ran Wang

Editor-in-Chief

Data in Brief

Dear Dr. Hao-Ran Wang:

This manuscript has not been published or presented elsewhere in part or in entirety and is not under consideration by another journal. We have rectified the manuscript, read and understood your journal’s policies, and we believe that neither the manuscript nor the study violates any of these. There are no conflicts of interest to declare.

Thank you in advance for your kind consideration of this paper.

Sincerely yours,

Muhamad Aidil Zahidin

Graduate Research Assistant

Institute of Tropical Biodiversity and Sustainable Development

Universiti Malaysia Terengganu

21030 Kuala Nerus

Malaysia

Tel: +6014-5861530

E-mail: [aiditarsier@gmail.com](mailto:aiditarsier@gmail.com), [aidil_germ@yahoo.com.sg](mailto:aidil_germ@yahoo.com.sg)
